# The Beat

**Published:** 2006-06

**Authors:** Erin E. Dooley

## New UNEP Leader Chosen

The UN General Assembly has unanimously selected Achim Steiner of Germany to succeed Klaus Töpfer as the fifth executive director of UNEP. Steiner is presently director-general of the World Conservation Union, the world’s largest environmental network, and will begin his four-year UNEP term in June 2006. Of the selection, Töpfer said, “I am convinced that choosing Achim Steiner will prove to be a great decision, bringing youth, dynamism, intellect, and a deeply held commitment to environment and sustainable development issues.” Steiner has degrees from Oxford University and the University of London, and has also studied at the German Development Institute and Harvard Business School.

**Figure:**
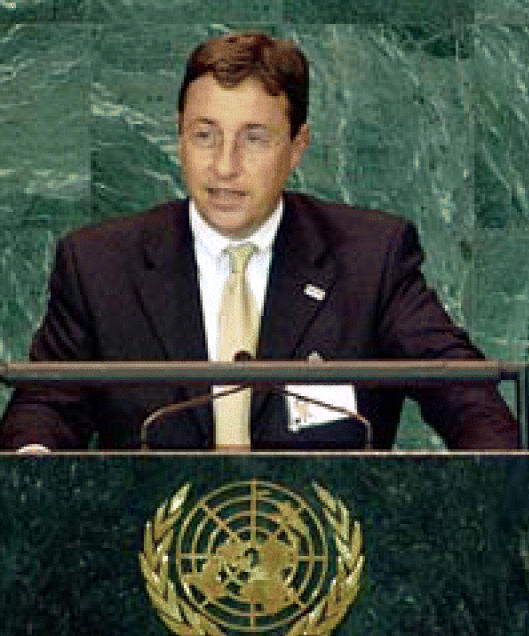


## China Approves Environment Plan

China is currently home to 16 of the world’s smoggiest cities, many of the country’s waterways are severely contaminated, and piles of construction refuse are being dumped in rural areas. Acid rain, industrial pollution, nuclear risks, and declining biodiversity also pose grave problems. In February 2006 China’s State Council approved a plan to combat the country’s pervasive pollution. A state media statement said, “The move is aimed at protecting the long-term interests of the Chinese nation and leaving a good living and development space for our offspring.” The plan calls for regional governments to set environmental targets to be evaluated regularly, and for local officials to be assessed on their environmental performance, not just their success in promoting economic development. Poor environmental performance by officials will be punishable under the plan.

## Safe Harbor for Fish Lovers

A number of recent public advisories have warned women of childbearing age to limit intake of swordfish, shark, tuna, and other fish with high levels of mercury, since studies show that brain development in young children is affected when their mothers consume such fish. Now northern Californian fish lovers who are concerned about mercury are in luck. Holiday Quality Foods markets and select Sam’s Club stores in the region now stock Safe Harbor certified fresh fish, which uses a new technology to measure the fish’s mercury content at the packaging plant in about one minute (conventional testing can take a week or more). Fish that register more than the median FDA level for that species are rejected. The certification is part of a test by the stores and Pacific Seafood Group, one of America’s largest fish wholesalers, to see if consumers would buy more fresh fish if they knew it contained safe levels of mercury.

**Figure:**
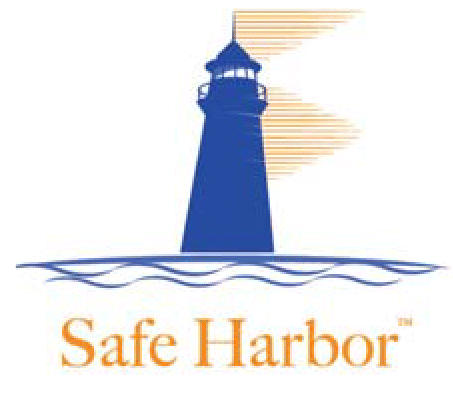


## America’s Best Development Projects

As part of its efforts to curb the expansion of low-density, vehicle-dependent communities, the Sierra Club has released its first *Guide to America’s Best Development Projects*. The guide spotlights 12 U.S. projects that the group holds up as models for building healthier and more sustainable communities—and invites local governments to demand more of them. The featured projects boast access to a range of transportation choices, redevelopment of existing urban areas, proximity of homes to shops and offices, preservation of existing community assets such as older buildings and natural resources, minimization of stormwater pollution and runoff, input from the local citizenry in planning, and use of green building methods.

**Figure:**
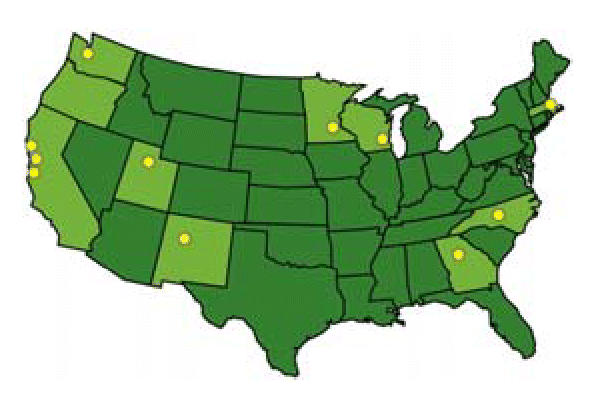


## Building Eco-Cities in China

The London-based consulting firm Arup has signed a multibillion-dollar contract with Chinese officials to design and build up to five self-sustaining “eco-cities” around China. The first eco-city, Dongtan, on an island near Shanghai, is expected to be home to about 1.15 million people by 2040. The city’s first phase, accommodating 50,000 residents, will be completed in time for the 2010 Shanghai Expo trade fair. Other locations have not yet been revealed. The cities, a magnet for investment funds, will feature the capture and purification of rainwater to support city life, use of organic waste materials as an energy source, and reduction of environmentally unfriendly landfills. Arup has also partnered with emissions brokerage CO2e to offset the carbon generated by personnel working on the initiative.

## Maine Mandates e-Recycling

In January 2006, Maine became the first state to require that TV and computer monitor makers pay for the cost of recycling their products once they are discarded by consumers. The state has approved five waste services to gather and sort the electronic waste, ship it to recyclers, and bill manufacturers according to the amount of waste they originated. Municipalities may choose whether they will operate an ongoing collection center, do regular one-day collections, or have their residents deliver items directly to a nearby consolidator.

**Figure:**
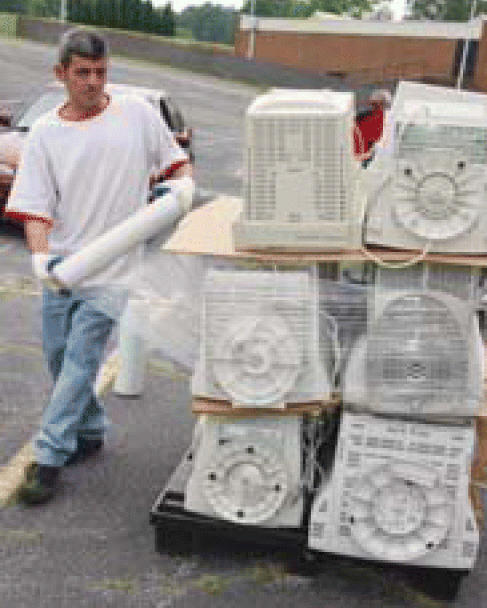


The law reflects similar initiatives in Europe and Japan to help keep toxic materials such as lead, mercury, cadmium, brominated flame retardants, and phosphorus coatings out of the environment. According to the EPA, e-waste is now the nation’s fastest-growing category of solid waste.

